# LATS1 exerts tumor suppressor functions via targeting Gli1 in colorectal cancer

**DOI:** 10.7150/jca.62211

**Published:** 2021-10-28

**Authors:** Zhengchao Shen, Yingying Pan, Peng Chen, Bin Jiang, Xiaosan Fang, Yaqi Jiang

**Affiliations:** 1 Department of Hepatobiliary Surgery, The First Affiliated Hospital of Wannan Medical College, Wuhu, Anhui, 241000, P.R. China.; 2 Department of Urinary Surgery, The Second Affiliated Hospital of Wannan Medical College, Wuhu, Anhui, 241000, P.R. China.

**Keywords:** LATS1, proliferation, migration, Gli1, colorectal cancer

## Abstract

**Background:** The Hippo pathway's primary kinase component, large tumor suppressor 1 (LATS1), has been hypothesized as a tumor suppressor in a variety of cancers. LATS1's biological effects on colorectal cancer (CRC) are yet to be determined.

**Methods:** The analysis of LATS1 mRNA expression in CRC was conducted using public databases from the Gene Expressing Profiling Interactive Analysis database (GEPIA). Investigation for the expression of LATS1 protein in 102 CRC tumor tissues and 57 normal tissues was performed using immunohistochemistry (IHC) analysis. *In vitro* genetic manipulation was used to explore the potential role and mechanism of LATS1 in the regulation of proliferation and migration of CRC cells.

**Results:** LATS1 was found to be considerably downregulated in CRC tissues, with much lower levels in individuals with bigger tumors of size (≥5 cm), deeper invasion (T3-4), positive lymph node metastasis (LNM), and advanced tumor-node-metastasis (TNM) stage (III-IV). As exhibited by clinical data analysis, LATS1 loss was significantly associated with TNM and LNM staging in CRC patients. Furthermore, our *in vitro* investigations revealed that LATS1 depletion increased CRC cell proliferation and migration in HCT116 cells, whereas overexpressing LATS1 had the opposite effect in SW620 cells. LATS1 suppressed the expression of glioma-associated oncogene-1 (Gli1), and LATS1's tumor-suppressive actions in CRC are dependent on Gli1. Moreover, LATS1 could modulate Yes-associated protein 1 (YAP1) expression and mTOR activation in CRC cells.

**Conclusion:** Our findings identify the LATS1 as a unique Gli1 regulator in CRC cell migration and proliferation, and suggest that LATS1 may serve as a potential therapeutic target for CRC.

## Introduction

Colorectal cancer (CRC) is one of the most common cancers worldwide, and it is still the third biggest cause of cancer-related mortality and morbidity 1-3. Despite the fact that individuals with CRC who are discovered early on have an overall survival rate of around 80%, over one-third of patients will die from their disease, primarily due to distant metastases 3. Emerging data confirm the involvement of several genes and cellular signaling pathways in the development and metastasis of CRC 4. Therefore, discovering new targets and clarifying the underlying biochemical pathways could lead to more effective CRC therapy techniques.

The Hippo pathway regulates various biological processes which include cell apoptosis, proliferation, and differentiation, to control tissue and organ size 5, 6. In mammalian systems, the core components of this pathway comprise the mammalian STE20-like protein kinase 1/2 (MST1/2), the large tumor suppressor 1/2 (LATS1/2), and their downstream transcription co-activators, Yes-associated protein 1 (YAP1) and TAZ 7, 8. MST1/2 kinase activates LATS1/2 by the process of phosphorylation, which leads to the phosphorylation of YAP1/TAZ and supplies a docking site for the 14-3-3 protein, causing cytoplasmic retention and ubiquitin-mediated proteasomal degradation of YAP1 and TAZ 5. Recently, accumulating evidence has suggested that perturbation of the Hippo pathway contributes to cancer development 5-9. LATS1, as the major kinase component of the Hippo pathway, exerts a significant role to control tumor cell proliferation, growth, and migration 10-13. It was suggested that it could be used as a tumor suppressor in a variety of cancers including breast carcinoma 14, 15, ovarian cancer 16, non-small cell lung cancer 17, hepatocellular carcinoma 18, cervical cancer 19 and gastric cancer 20. In CRC, LATS1 protein expression was downregulated and its decreased expression was associated with promoter hypermethylation 21. The exact functional significance and fundamental mechanisms of LATS1 in CRC, however, are unknown and need to be investigated.

The Hedgehog signaling system is known to be involved in the genesis and progression of malignancies, including CRC 22, 23. Smoothened (SMO), known as the canonical Hedgehog pathway, can activate Gli1, a major transcription factor in the Hedgehog signaling pathway 24. It can also be activated by protein kinase B (AKT), extracellular signal-regulated kinase (ERK), and mammalian target of rapamycin (mTOR) which is known as the non-canonical Hedgehog pathway 24, 25.

Herein, LATS1 expression in CRC tissues was investigated, as well as the relationship between LATS1 protein expression and clinicopathological variables. LATS1's biological roles in CRC were also investigated using the loss of function and gain of function studies. Moreover, our findings revealed new mechanical insights into the critical functions of LATS1 in reducing Gli1 via modulating YAP1 expression and mTOR activation, potentially implying new CRC treatment options.

## Materials and methods

### Clinical data and tissue samples

The relevant clinical data were gathered from the Gene Expressing Profiling Interactive Analysis database (GEPIA) which can be found at http://gepia.cancer-pku.cn/. Formalin-fixed and fresh tissue samples were acquired from Wannan Medical College's First Affiliated Hospital and verified by pathological diagnosis. Prior to surgery, none of the patients had chemo or radiotherapies. This study was approved by the Institute Research Medical Ethics Committee of Wannan Medical College's First Affiliated Hospital. In compliance with the Declaration of Helsinki, all subjects supplied written informed consent. Table 1 summarizes the characteristics of the patients.

### Immunohistochemistry (IHC)

IHC staining of paraffin-embedded human tissues was carried out as reported previously 25, 26. In brief, Samples were dewaxed, rehydrated and endogenous peroxidase activity was blocked with 3% hydrogen dioxide. The sections were incubated with the primary antibodies recognizing human LATS1 (dilution 1:100; #17049-1-AP, Proteintech) overnight at 4 °C, and with the secondary antibody for 1 hour at room temperature. Finally, the sections were visualized using a tissue staining kit (Zhongshanjinqiao Biotechnology, Beijing, China). LATS1 protein expression were scored according to the percentage of positive cells and the staining intensity. Percentage score was assigned as 1 (<25%), 2 (25-50%), 3 (51-75%), 4 (76-100%). The staining intensity was graded into 0 (no staining), 1 (week), 2 (moderate), and 3 (strong). A final score was calculated by the multiple of staining percentage and intensity. The LATS1 protein levels were considered as high when the final scores were 5 or above, and as none or low expression when the final scores were < 5.

### Cell culture and transfection

Chinese Academy of Sciences Committee Type Culture Collection cell bank (Shanghai, China) was provided the human CRC cell lines (HCT8, HCT116, LoVo, RKO, SW480, SW620, CaCo2, and DLD-1). We maintained the cells in 10% fetal bovine serum (FBS) supplemented RPMI 1640 medium (Gibco, USA) containing 1% penicillin/streptomycin (P/S; Gibco, USA) at 37 °C with a 5% CO_2_ atmosphere. The Lentivirus vectors carrying LATS1 overexpression or short hairpin RNA (shRNA) targeting LATS1 and their control vectors were invented by Genechem Company (Shanghai, China). We performed the transfection following the company's instructions, which were documented in our earlier study 25, 26. Lipofectamine RNAiMax (Invitrogen) was used to transfect small interfering RNA (siRNA) at 20 nM of final concentration, as directed by the manufacturer. The shRNA sequence targeting human LATS1 is 5′-ATCCTCGACGAGAGCAGA-3′. The siRNA sequence targeting human Gli1 is 5′-CUCCACAGGCAUACAGGAU-3′.

### Western blot analysis and Protein extraction

RIPA lysis buffer (Beyotime, China) was used for the extraction of total proteins, and the determination of protein content was carried out with a BCA protein assay kit (Pierce, USA). The protein separation was performed on an 8-10% polyacrylamide gel by sodium dodecyl sulphate polyacrylamide gel electrophoresis (SDS‐PAGE) and then transferred to polyvinylidene difluoride (PVDF) membranes. Chemiluminescence was used to visualize the proteins after they had been incubated with secondary and primary antibodies. The bands were quantified using the Imaging J analysis software. The detailed protocol was described previously 25, 26. Following antibodies used in this study: anti-LATS1 (dilution 1:1000; #17049-1-AP, Proteintech), anti-Gli1 (dilution 1:1000; #ab217326, Abcam), anti-YAP1 (dilution 1:1000; #14074, Cell Signaling Technology (CST)), anti-p-mTOR (dilution 1:1000; #9205, CST), anti-mTOR (dilution 1:1000; #2983, CST), anti-GAPDH (dilution 1:5000; #AG019, Beyotime).

### RNA extraction and quantitative real-time PCR (qRT-PCR)

Total RNA was prepared as per the manufacturer's instructions using TRIzol reagent (Thermo Fisher Scientific). cDNA was synthesized and amplified by PCR using specified primers. qPT-PCR was performed on ABI 7500 Fast Real-Time PCR System (Applied Biosystems, UK) with SYBR Green RT-PCR kits (ABI, USA). As an endogenous control, the human GAPDH were used. The study used the following primers: human-LATS1-Forward, GTT AAG GGG AGA GCC AGG TCCTT, human-LATS1-Reverse, TCA AGG AAG TCC CCA GGA CTGT; human-GAPDH-Forward, AAT CCC ATC ACC ATC TTC, human-GAPDH-Reverse, AGG CTG TTG TCA TAC TTC 26.

### Assay for migration and cell viability

Colony formation and Transwell migration assays were used to identify cell proliferation and migration ability respectively by using the protocol described previously 25, 27.

### Statistical analysis

Means ± standard errors of the mean (SEM) are used to express the data. The experimental results were statistically evaluated using two-tailed Student's* t*-test or one-way analysis of variance (ANOVA). The chi-square statistical test or Fisher's exact test was used to examine the IHC results. For all tests, a *P*-value of < 0.05 was considered statistically significant.

## Results

### Expression of LATS1 is decreased in human CRC tissues

**Figure 1:**
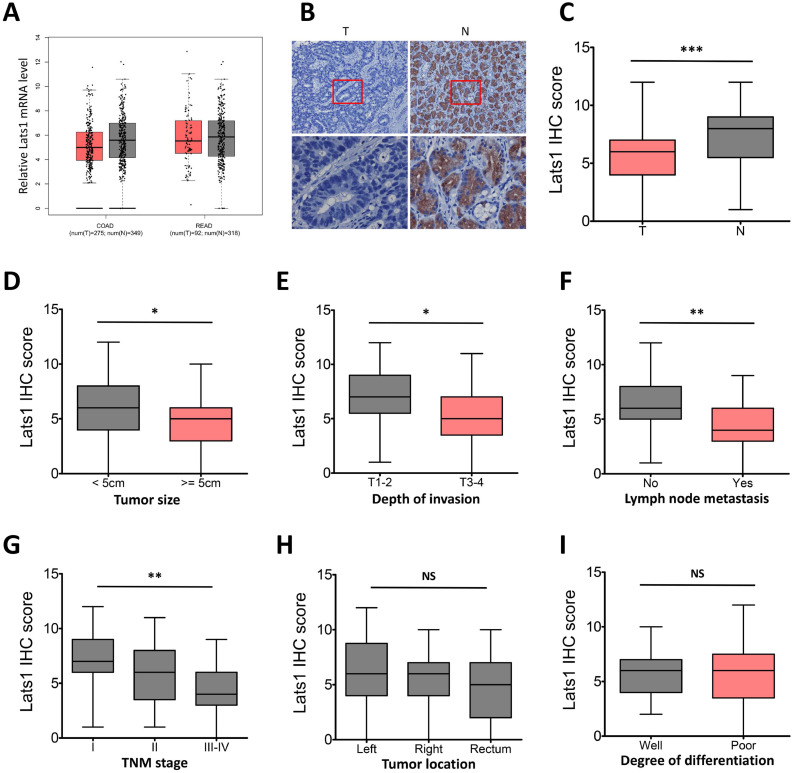
** LATS1 expression in human CRC tissues. (A)** Data from the GEPIA database showed that LATS1 mRNA was downregulated in CRC tumor tissues (T) compared to normal controls (N), COAD colon adenocarcinoma, READ rectal adenocarcinoma. **(B)** IHC staining for LATS1 in CRC tissues and peritumoural normal tissues. **(C)** The IHC score of LATS1 in CRC tissue samples (T) and normal tissues (N). **(D-I)** LATS1 expression in CRC tumor tissues of different tumor size (**D**), depth of invasion (**E**), lymph node metastasis (**F**), TNM stage (**G**), tumor location (**H**) and degree of differentiation (I). NS, nonsignificant, *P < 0.05, **P < 0.01, ***P < 0.001.

We first investigated the mRNA expression of LATS1 via the GEPIA database, and the results showed decreased mRNA level of LATS1 in CRC tumor tissues (T) compared to the normal controls (N, Figure 1A). To further determine the role of LATS1 in colorectal tumorigenesis, an IHC assay was conducted in human para-cancer normal and CRC tissues. IHC staining revealed that the positive LATS1 expression rates were 82.5% (47/57) in normal colorectal tissues and 63.7% (65/102) in CRC tissue samples (Figure 1B, Table 1). When compared to normal tissue samples, the IHC score of LATS1 staining in CRC tissue samples was lower (*P* < 0.001, Figure 1C). Furthermore, LATS1 protein expression levels in CRC tumor tissues with larger tumor sizes (≥5 cm), deeper invasion (T3-4), positive lymph node metastasis (LNM), or advanced tumor-node-metastasis (TNM) stage (III-IV) were lower significantly as compared to control groups (Figure 1D-G). However, there was no link found between LATS1 protein expression and tumor site or differentiation level (Figure 1H and I).

The relationship between LATS1 protein expression and clinicopathological features in CRC tumor tissues was then investigated further. Chi-square test analysis showed the negative correlation of LATS1 protein expression with LNM (*P* = 0.001) and TNM staging (*P* = 0.002), but no correlation was observed with other clinicopathological factors such as tumor size, gender, age, tumor location, depth of invasion and degree of differentiation (*P* > 0.05, Table 1).

### LATS1 expression in human CRC cell lines

**Figure 2:**
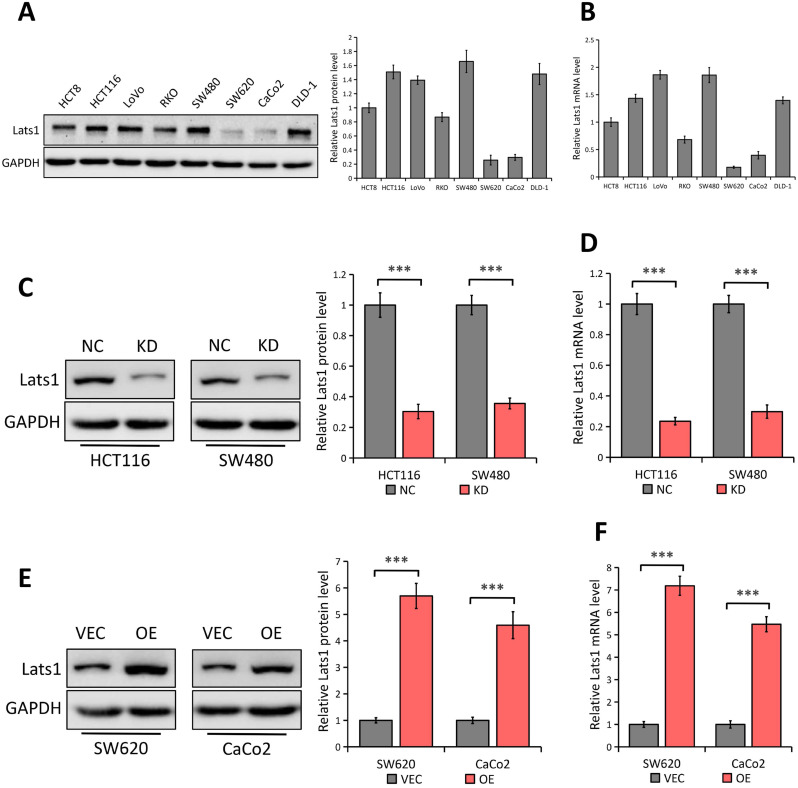
** LATS1 expression in human CRC cell lines. (A-B)** Western blot (A) and qRT-PCR (B) analysis of LATS1 expression in CRC cell lines (HCT8, HCT116, LoVo, RKO, SW480, SW620, CaCo2 and DLD-1). **(C-D)** Western blot (C) and qRT-PCR (D) analysis of LATS1 expression in HCT116 and SW480 cells stably transfected with control-shRNA (NC) or shRNA against LATS1 (KD). **(E-F)** Western blot (E) and qRT-PCR (F) analysis of LATS1 expression in SW620 and CaCo2 cells stably transfected with empty vector (VEC) or plasmids encoding human LATS1 (OE). ***P < 0.001.

By using western blot and qRT-PCR analyses, we detected the LATS1 expression in eight human CRC cell lines (HCT8, HCT116, LoVo, RKO, SW480, SW620, CaCo2, and DLD-1) (Figure 2A and 2B respectively). Compared with the low expression of LATS1 at both mRNA and protein levels in SW620 and CaCo2 cell lines, other cell lines (especially HCT116 and SW480) showed a relatively high concentration of LATS1 (Figure 2A and B). Hence, we used a lentivirus-based shRNA approach to knock down LATS1 expression in HCT116 and SW480 cells, which had significant amounts of endogenous LATS1 protein and mRNA. LATS1 expression was lesser in cells transfected with LATS1-targeting shRNA (KD) than in cells transfected with control-shRNA (NC, *P* < 0.001) (Figure 2C and 2D representing the result of Western blot and qRT-PCR respectively). Similarly, we used both analyses to examine the overexpression effectiveness of human LATS1 encoded with plasmid or empty vector in SW620 and CaCo2 cells that produced relatively low endogenous LATS1 (Western Blot, Figure 2E) and (qRT-PCR, Figure 2F). The results showed that cells transfected with plasmids containing human LATS1 (OE) expressed more LATS1 protein and mRNA than cells transfected with empty vector (VEC, *P* < 0.001).

### LATS1 inhibits the proliferation and migration of CRC cells

**Figure 3:**
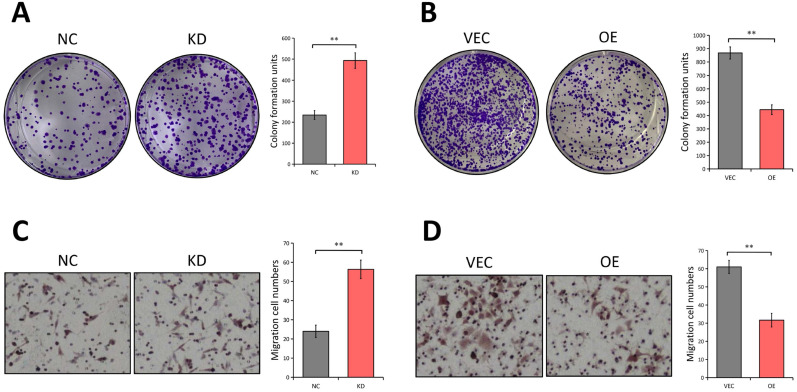
** LATS1 plays tumor-suppresive roles in CRC. (A)** Colony formation assay in HCT116 cells stably transfected with control-shRNA (NC) or shRNA against LATS1 (KD). **(B)** Colony formation assay in SW620 cells stably transfected with empty vector (VEC) or plasmids encoding human LATS1 (OE). **(C)** Transwell assay in HCT116 cells (NC vs. KD). **(D)** Transwell assay in SW620 cells (VEC vs. OE). **P < 0.01.

Since the LATS1 expression was reduced in CRC tissues, we next investigated the potential biological functions of LATS1 in CRC cells. A colony formation experiment was used to see if LATS1 influences the proliferation of CRC cells. The results revealed that the colony number of HCT116 cells dramatically increased following depletion of LATS1 (*P* < 0.01, Figure 3A), while the foci forming potential of SW620 cells was significantly reduced under elevated LATS1 level (*P* < 0.01, Figure 3B). We then used a transwell test to see how LATS1 affected cell migration. The analysis indicates that reducing LATS1 amplified the capacity of HCT116 cells to migrate (*P* < 0.01, Figure 3C). In contrast to the control group, HCT116 cells with stable LATS1 overexpression demonstrated a significant reduction in migratory cells (*P* < 0.01, Figure 3D).

### The tumor-suppressive function of LATS1 is dependent on Gli1

**Figure 4:**
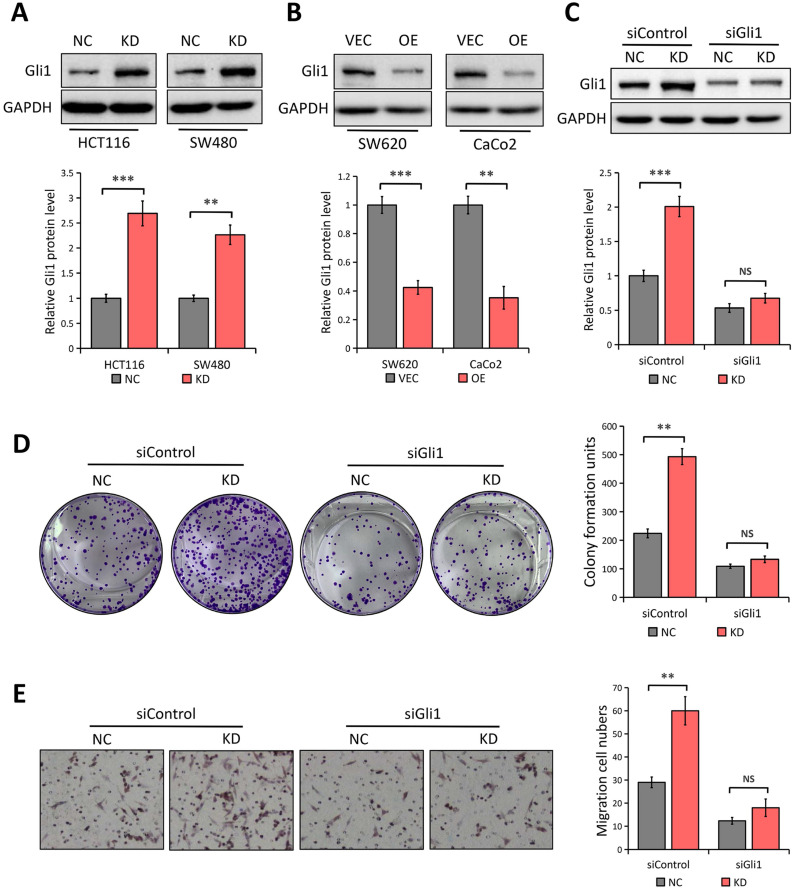
** LATS1 inhibits CRC cell proliferation and migration in a Gli1-dependent manner. (A)** Western blot analysis of Gli1 expression in HCT116 and SW480 cells stably transfected with control-shRNA (NC) or shRNA against LATS1 (KD). **(B)** Western blot analysis of Gli1 expression in SW620 and CaCo2 cells stably transfected with empty vector (VEC) or plasmids encoding human LATS1 (OE). **(C)** Western blot analysis of Gli1 expression in LATS1-depleted HCT116 cells with or without Gli1 siRNA treatment. **(D)** Colony formation assay in LATS1-depleted HCT116 cells with or without Gli1 siRNA treatment. **(E)** Transwell assay in LATS1-depleted HCT116 cells with or without Gli1 siRNA treatment. NS, nonsignificant, **P < 0.01, ***P < 0.001.

We predicted that Gli1 might be involved in LATS1-mediated inhibition of CRC cell to proliferate and migrate, given that abnormal Gli1 expression in the Hedgehog pathway promotes CRC development 28, 29. LATS1 knockdown enhanced the expression of Gli1 in SW480 and HCT116 cells (*P* < 0.01; Figure 4A). Conversely, the levels of Gli1 protein were significantly decreased in LATS1-overexpressing cells (*P* < 0.01, Figure 4B) indicating the suppression of Gli1under the influence of LATS1 in CRC cells.

We employed Gli1-specific siRNA (siGli1) to knock down the expression of Gli1 in HCT116 cells with or without LATS1 depletion to see if Gli1 is involved in LATS1-mediated reduction of cell proliferation and migration in CRC. According to the Western blot analysis, the Gli1 protein level was decreased in cells transfected with siGli1 as compared to the control group (Figure 4C). In colony formation and transwell migration assays, LATS1 knockdown considerably boosted the proliferation and migratory ability of HCT116 cells (*P* < 0.01, Figure 4D and E), but the enhanced proliferation and migration was greatly impaired when Gli1 was silenced (*P* > 0.05).

### LATS1 regulates YAP1 expression and mTOR activation in CRC cells

**Figure 5:**
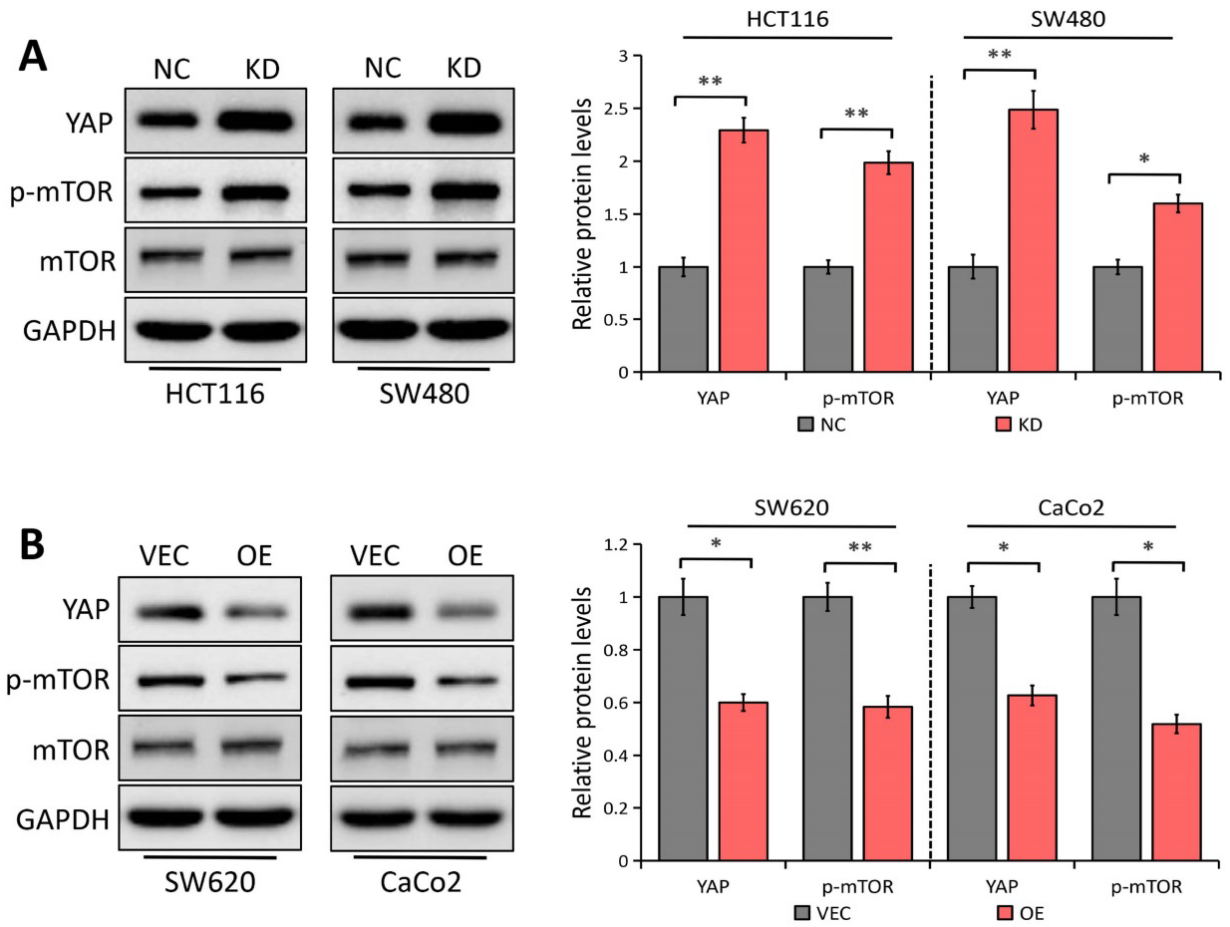
** LATS1 regulates YAP1 expression and mTOR signaling pathway. (A)** Western blot analysis of the indicated proteins in HCT116 and SW480 cells stably transfected with control-shRNA (NC) or shRNA against LATS1 (KD). **(B)** Western blot analysis of the indicated proteins in SW620 and CaCo2 cells stably transfected with empty vector (VEC) or plasmids encoding human LATS1 (OE). *P < 0.05, **P < 0.01.

In esophageal squamous cell carcinoma, YAP1 has been shown to upregulate Gli1 30, which is in accordance with our previous study in gastric cancer 30, representing that YAP1 enhances cell migration and proliferation via regulating Gli1 expression through the AKT/mTOR signaling pathway. It was also reported that LATS1 suppresses mTOR activity 15 and activated mTOR can enhance Gli1 expression in an SMO-independent manner 24. Considering LATS1 as a critical regulator of the YAP1 signaling 5, we speculated that YAP1/mTOR signaling might be implicated in LATS1 inhibition of Gli1. The Western blot analysis showed that LATS1 depletion notably promoted YAP1 expression and mTOR phosphorylation in both SW480 and HCT116 cells (*P* < 0.05, Figure 5A), with no effect on the total mTOR level. On the contrary, LATS1 elevation inhibited YAP1 expression and mTOR activity in SW620 and CaCo2 cells (*P* < 0.05, Figure 5B). Our findings suggest that LATS1 regulates the YAP1/mTOR signaling pathway in CRC cells, which could explain the reason for Gli1 suppression.

## Discussion

The Hippo pathway's primary kinase component, LATS1, has been hypothesized as a tumor suppressor in a variety of cancers 14-20. LATS1 protein expression was downregulated in CRC, and this was linked to promoter hypermethylation 21, although the specific functional aspects and underlying mechanisms of LATS1 in CRC remain unknown.

In this work, we evidenced the downregulation of LATS1 expression in human CRC which was reduced further in patients with positive LNM, and advanced TNM stage (III-IV). Furthermore, clinical data analysis revealed that loss of LATS1 protein expression has a significant association with TNM and LNM staging in CRC patients. Moreover, *in vitro* gain of function and loss of function tests revealed that silencing LATS1 dramatically increased the CRC cell's potential to proliferate and migrate, whereas LATS1 overexpression did the opposite, signifying the tumor-suppressive effects of LATS1.

Gli1, as a key transcription factor of the Hedgehog signaling pathway, exerts a critical role in CRC development 28, 29. Our findings imply that LATS1 regulates Gli1 expression in CRC, as authenticated by the fact that LATS1 knockdown enhanced the expression of Gli1 while LATS1 elevation did the opposite. Gli1 was silenced in HCT116 cells with or without LATS1 depletion to see if it was involved in LATS1 regulation of CRC cell proliferation and migration and it was observed that LATS1 knockdown promoted the migration and proliferation of HCT116 cells, but the enhanced proliferation and migration ability was greatly impaired when Gli1 was depleted, indicating the dependence of anti-carcinogenic roles of LATS1 on Gli1.

Given the facts that LATS1 is a critical regulator of YAP1 5 and YAP1 can upregulate Gli1 expression in esophageal squamous cell carcinoma 30 and gastric cancer 31, we speculated that YAP1 might be implicated in LATS1-mediated Gli1 suppression. Here, we confirmed that LATS1 depletion promoted YAP1 expression while LATS1 elevation inhibited YAP1 expression in CRC cells. Furthermore, it was recently discovered that LATS1 reduces mTOR activity to directly coordinate the mTORC1 and Hippo pathways in growth control 15 and activated mTOR can enhance Gli1 expression 24, we asked whether the mTOR signaling is involved in LATS1-regulated Gli1 inhibition. The results showed that LATS1 depletion notably promoted mTOR phosphorylation in both HCT116 and SW480 cells, without affecting the expression level. On the contrary, LATS1 elevation inhibited mTOR activity in SW620 and CaCo2 cells. Moreover, another possible mechanism is that LATS1 elevation decreased YAP1 protein level, leading to mTOR inactivation, which subsequently suppressed Gli1 expression. Collectively, findings suggest that LATS1 works as a regulator of the YAP1/mTOR signaling pathway in CRC cells, and is capable to cause Gli1 suppression.

Although more in-depth studies are needed to clarify the mechanisms regarding the Gli1 regulation by LATS1 in CRC, our results strongly confirmed that the LATS1 is a tumor suppressor in CRC. Furthermore, we discovered a putative role for LATS1 to regulate the CRC cell proliferation and migration by direct or indirect inhibiting Gli1. This could open up a new avenue for CRC treatment by targeting the LATS1/Gli1 signaling axis.

## Figures and Tables

**Table 1:** Relationship between LATS1 protein expression (detected by IHC) and clinic-pathological factors in 102 CRC patients

Clinic parameters	Case No.	LATS1 expression		χ^2^	*P* value
		None or low	High		
**Total**	102	37	65		
**Age (years)**					
<65	37	15	22	0.457	0.499
≥65	65	22	43		
**Gender**					
Male	68	22	46	1.357	0.244
Female	34	15	19		
**Tumor size**					
<5 cm	53	15	38	3.034	0.082
≥5 cm	49	22	27		
**Tumor location**					
Left-sided colon	32	12	20	1.955	0.376
Right-sided colon	33	9	24		
Rectum	37	16	21		
**Degree of differentiation**					
Well	89	32	57		1.000
Poor	13	5	8		
**Depth of invasion**					
T1-2	17	3	14	3.062	0.080
T3-4	85	34	51		
**Lymph node metastasis**					
Yes	42	23	19	10.557	0.001**
No	60	14	46		
**TNM stage**					
I-II	59	14	45	9.529	0.002**
III-IV	43	23	20		
